# Staggered spondaic word test in epileptic patients

**DOI:** 10.1590/S1516-31802002000600006

**Published:** 2002-11-01

**Authors:** Karin Zazo Ortiz, Liliane Desgualdo Pereira, Alda Christina Lopes de Carvalho Borges, Luiz Celso Pereira Vilanova

**Keywords:** Epilepsy, Auditory Perception, Central, Auditory, Processing, Epilepsia, Percepção Auditiva, Processamento, Auditivo, Central

## Abstract

**CONTEXT:**

Auditory processing during childhood may be altered if there is any predisposing factor during the course of development. Neurological disorders are among the risk factors for auditory processing impairment. From this perspective, epileptic children present such a risk factor and could present auditory processing dysfunction.

**OBJECTIVE:**

To evaluate central auditory processing in epileptic patients using the *Staggered Spondaic Word Test (SSW)* in order to verify whether these patients presented auditory disorders and whether the type of crisis – partial or generalized – played a role in the occurrence and type of disorder.

**SETTING:**

Tertiary care hospital.

**SAMPLE:**

Thirty-eight children and adolescents, ranging from 7 to 16 years old, with a diagnosis of epilepsy divided into two groups: 23 patients with partial crisis and 15 patients with generalized crisis.

**MAIN MEASUREMENTS:**

Performance in the Staggered Spondaic Word Test versus epileptic crisis type (partial or generalized).

**RESULTS:**

The majority of epileptic patients showed central auditory processing disorders. There were no differences in relation to crisis type. Both groups showed similar performance, although the results observed for these patients differ from what is obtained with normal populations. With regard to response bias, there were also no differences in performance between subjects with partial or generalized seizures. All possible disorders were found in both groups, without the prevalence of one specific disorder over the other.

**CONCLUSIONS:**

This study revealed a high prevalence of disorders among epileptic patients in relation to processing partially overlapped verbal sounds in a dichotic paradigm.

## RESUMO

**CONTEXTO:**

O processamento da informação auditiva na infância pode ser alterado se hou-ver algum fator predisponente durante o de-senvolvimento. As alterações neurológicas são um fator de risco para distúrbios no processamento da informação auditiva. Nesta perspectiva, crianças com epilepsia teriam um fator de risco e poderiam apresentar alte-rações do processamento auditivo.

**OBJETIVO:**

Avaliar o processamento auditivo central de pacientes epilépticos por meio do teste Staggered Spondaic Word Test (SSW), a fim de verificar se estes pacientes apresen-tavam alteração e se o tipo de crise ¾ parcial ou generalizada ¾ interferiria na ocorrência e no tipo da desordem.

**LOCAL:**

Universidade Federal de São Paulo e Hospital Israelita Albert Einstein.

**AMOSTRA:**

38 crianças e adolescentes, na faixa etária compreendida entre 7 e 16 anos, com diagnóstico neurológico de epilepsia, reunidos em dois grupos: um formado por 23 pacientes que apresentavam crise parcial e ou-tro formado por 15 pacientes que apresenta-vam crise generalizada.

**VARIÁVEIS ESTUDADAS:**

Desempenho dos indivíduos no teste dicótico de dissílabos alternados (Staggered Spondaic Word Test ¾ SSW) *x* tipo de crise epiléptica ¾ parcial ou generalizada.

**RESULTADOS:**

A maior parte dos pacientes com epilepsia apresentou alteração do proces-samento auditivo central, identificadas no teste SSW. Não houve diferenças em relação ao tipo de crise, generalizada ou parcial, tendo ambos os grupos apresentado desempe-nho similar, porém diferente do encontrado na população normal. Em relação às tendên-cias de erros, também não foram observadas diferenças de desempenho, entre os indiví-duos com crise epiléptica parcial ou generalizada, tendo sido encontrados todos os tipos de alterações, sem se caracterizar a ocorrên-cia de uma alteração específica para algum dos grupos estudados.

**CONCLUSÕES:**

Neste estudo ficou demonstrada a alta prevalência de alterações no processamento dos sons verbais parcialmente sobrepostos em tarefa dicótica em pacientes epilépticos.

## INTRODUCTION

Central auditory processing assessment has been used as a diagnostic tool for almost 50 years. The dichotic listening paradigm was introduced by Broadbent (1954)^1^ and, following Kimura's first studies (1963),^2^ a large volume of research has been produced. Processing of auditory information during childhood may be altered if any predisposing factor is present during the course of development.

Neurological disorders are risk factors for dysfunction in the processing of auditory information termed central auditory processing (CAP).^3,4^ From this perspective, epileptic children present such a risk factor and could present central auditory processing disorders. The majority of research related to epilepsy dysfunction is based on the study of cognitive or linguistic abilities. These studies reveal that the cognitive performance of epileptic children differs in comparison with control groups and it also differs between groups of epileptic children when different crisis types are studied: partial or generalized crises.^5-7^

In view of the fact that cognitive functions involve attention and memory and that central auditory processing also involves attention and memory, we suspected that there could be performance differences in tests assessing CAP in epileptic patients with partial or generalized crises.

Since various mechanisms are analyzed through different procedures during CAP evaluation, we selected one of them for this study: the recognition of overlapped sounds presented dichotically, using the *Staggered Spondaic Word Test* (SSW) in Portuguese.

The purpose of this study was to verify the performance of epileptic children in the SSW Test, in order to establish whether these patients presented disorders and whether crisis type –partial or generalized – played a role in the occurrence, type and nature of dysfunction.

## METHODS

This study was carried out at the Hearing Disorders Clinic of the Otolaryngology and Human Communication Disorders Department at Universidade Federal de São Paulo –Escola Paulista de Medicina and at the Speech Pathology and Audiology Clinic of Hospital Israelita Albert Einstein in São Paulo, Brazil.

Thirty-eight children and adolescents, ranging from 7 to 16 years old, with a neurological diagnosis of epilepsy, were evaluated. Patients were divided into two groups: 23 patients presenting partial epilepsy and 15 patients presenting generalized crisis. All subjects had Brazilian Portuguese as their mother tongue. The patients were utilizing either a single drug or combined therapy.

All patients underwent pure tone audiometry and acoustic emittance measurements. Illiterate patients whose hearing thresholds were outside of normal limits and patients with brain lesions confirmed via imaging tests (computerized tomography or magnetic resonance imaging) were excluded from the study group.

The subjects thus selected underwent the *Staggered Spondaic Word Test* (SSW) in Portuguese, i.e. a staggered dichotic word test. SSW test results from normal populations studied by Santos (1993)^8^ and Câmara (1998)^9^ were used as the control groups for our study.

This test was first introduced by Katz in 1962^10^ and was adapted for use in Portuguese by Borges (1986).^11^ It aims at evaluating binaural integration through a dichotic listening task involving pairs of disyllabic words, presented to each ear in an overlapping fashion. The ear receiving the first disyllabic word is alternated as the test stimuli are alternated between the two channels of the audiometer. The patient is asked to reproduce the group of words heard, while maintaining the presentation order. Partial overlapping refers to the second word of the first pair presented in one ear and the first word of the second pair presented in the opposite ear.

This test was designed using 80 pairs of disyllabic words, totaling 160 verbal stimuli. These 80 word pairs were presented at 50 dB HL, organized in sets of four words each. The patient was asked to hear the four-word group presented in one or both ears and then to reproduce them. Of 80 word pairs, 40 were first presented in the right ear and 40 in the left ear.

The word pairs were tape-recorded and presented using a MAICO MA-22 audiometer coupled either to a CD 2300 Dolby Auto Stop stereo cassette tape recorder, or using a Midimate 622 audiometer coupled to a Sony CD player.

A quantitative analysis was carried out, in which the total number of errors was scored for each subject and for each condition, in columns. The results were recorded using a specific protocol divided into columns, taking into consideration two criteria: the ear where the first test item was presented and the presentation condition. As we had two ears and four presentation conditions, there were eight columns: a, b, c, d, e, f, g and h, using the right ear as the reference. In this study, the wrong answer was not analyzed in relation to omission or repetition. Qualitative analysis regarding response bias was carried out considering reversals, ear effect, order effect and type A pattern, defined as follows:

- Reversals are responses in which the word order of an item is changed and there is no more than one mistake in the item.
- Ear effect refers to making a greater number of mistakes when the test begins in one specific ear rather than the other: in the right ear (ear effect high/low) or in the left ear (ear effect low/high).
- Order effect refers to making more mistakes involving the first or the last two words of an item. If there are significantly more errors at the beginning than at the end of the item, this is called a high/low order effect and, on the other hand, if there are more errors at the end, this is called a low/high order effect.
- Type A Pattern refers to a peak of mistakes occurring in the second word, both when the test begins in the right and in the left ear.

After data collection, appropriate statistical tests were applied. The significance level was considered to be 5% (or p ≤ 0.05).

## RESULTS

The first comparison made was between the total number of errors in the SSW test among epileptic children with partial seizures (mean = 32.3) and the total number of errors among epileptic children with generalized crises (mean = 37.5). It was seen that there was no statistical difference between these groups. Average values such as median, mean and standard deviation, minimum and maximum error values were then studied for each test condition. The Mann-Whitney test, comparing the number of errors in the two groups under each test condition (Table 1) did not reveal any significant difference between the groups for any of the test conditions. Both when considering the overlapping versus non-overlapping conditions and considering the total number of errors, we found a high rate of disorders in the SSW Test for both groups of epileptic patients studied.

**Table 1:** Descriptive values (median and P95-P5) and minimum and maximum number of errors for each column of the SSW and Mann-Whitney test results, comparing the number of errors for each group under different test situations

	A		B		C		D		E		F		G		H	
	Partial	Generalized	Partial	Generalized	Partial	Generalized	Partial	Generalized	Partial	Generalized	Partial	Generalized	Partial	Generalized	Partial	Generalized
Median	0	1	4	6	4	6	3	3	2	1	5	5	4	5	3	2
Maximum value	4	10	16	17	14	11	8	10	9	10	14	15	11	14	8	9
Minimum value	0	0	0	0	0	1	0	0	0	0	1	0	0	0	0	0
P5	0	0	0	0	0.2	1	0	0	0	0	1	0	0.2	0	0	0
P95 / P90	6.4	7	16	15.8	14	10.4	8	9.4	8.2	7	14.8	15	11	11.6	7.8	9
P95-P5 / P90-P5 difference	6.4	7	16	15.8	13.8	9.4	8	9.4	8.2	7	13.8	15	12.8	11.6	7.8	9
**Mann-Whitney test (partial vs. generalized crisis)**	**0.0720**		**0.3145**		**0.8338**		**0.9399**		**0.9392**		**0.5578**		**0.6097**		**0.9759**	

P = percentile.

Patients with partial crises presented similar results in relation to the number of errors under overlapping and non-overlapping conditions. This occurred for almost all comparisons, except when comparing right-ear non-overlapping conditions at the beginning (Column A) and at the end of the word group (Column H). These patients showed a greater number of errors in the last word than in the first one.

When analyzing the results from subjects with partial or generalized crises and considering the predominance of impairment in processing dichotic information from the right ear, left ear or both, we noted that there was no predominant disorder of one group over any other.

A comparison of subjects with partial or generalized crisis in relation to qualitative types of disorder in the SSW test (Table 2) showed that there was no predominance of any qualitative disorder for either group. This was true even when both groups of epileptic patients were considered as a whole, although a greater number of low/high order effects were observed, albeit without statistical significance. In analyzing qualitative disorders in relation to crisis type, we could observe a high prevalence of disorders in both groups. Nineteen out of 23 patients with partial crises and 14 out of 15 patients with generalized seizures presented qualitative disorders in the SSW test.

**Table 2:** Distribution of patients with partial or generalized crisis according to the qualitative disorder in the Staggered Spondaic Word Test

		Partial crisis (n = 23)	Generalized crisis (n = 15)
**Disorder**	Low/high ear effect	3	2
	High/low ear effect	4	1
	Low/high order effect	9	7
	High/low order effect	1	4
	Type A pattern	2	2
	Reversals	6	4
**No disorder**		4	1

## DISCUSSION

In this study, we observed a high prevalence of errors in processing verbal sounds that were partially overlapped (dichotic paradigm) among epileptic patients. The disorder may be related to failure in the functional integration of areas such as the brain stem, thalamus, hippocampus and corpus callosum and cortical areas such as the frontal, temporal and associated areas.

We believe that structural, histopathological and biochemical dysfunction that is usually associated with blood flow disorders in epileptic patients could explain the difference in stimulus processing between epileptic patients and normal subjects.

Histopathological studies have revealed hypometabolic areas at the focus of epileptic crises,^12,13^ with synapse and neuronal activity losses in the presence of disorders arising from epilepsy. According to several authors,^14-20^ dendritic and neuronal losses are always present in epileptic patients. These losses are independent of crisis type, and if we consider the anatomical and histopathological disorders responsible for poor performance by epileptic patients in the auditory skills evaluated in the SSW test, these authors’ findings would explain such performance observed in both groups of epileptic patients studied here.

Structural and biochemical alterations may occur at different localities along the auditory pathway, leading to a high incidence of disorders that do not differ functionally and are usually diffuse. Musiek & Pinheiro (1987)^21^ stated that an accurate determination of lesion locations and their depth and extent is usually impossible. Moreover, no single lesion or dysfunction would be capable of damaging the auditory function assessed in one specific test. Many different areas would be involved in the processing of verbal and nonverbal stimuli. A verbal response would require a decoding route from subcortical regions of the posterior temporoparietal areas through intra-hemispheric tracts of white matter to the frontal regions close to or in the central sulcus, where a motor response could be organized and initiated. Hence, the corpus callosum with its fibers of cortical projection would also play a definite role in sound processing and response production. There are some studies with epileptic patients indicating that such patients may exhibit subtle difficulties in organizing speech motor gestures.^22,23^ This would especially involve activities such as maintenance of speech velocity, phonological errors, production of sequences of complex syllables or words, which could also be considered as variables.

In addition, the interference of either attentional or recent memory factors could be an explanation for why patients with partial crisis showed a greater number of errors in the last words than in the first ones. Different authors have reported poor performance in recent memory tasks involving failures of verbal processing, information storage or retrieval or failures just in the initial information processing.^24^ These authors believe that the frequent occurrence of minimal electrical discharge may damage memory functioning during the stages of registration and consolidation of new information. This difficulty is probably related to the hippocampus region or it might be directly related to the proximity of the focus for primary areas and associated cortex.^25-27^

No studies dealing with psychoacoustic audiometric tests within auditory processing evaluation of epileptic patients were found in the literature. All the studies involving the auditory processing of epileptic patients were performed using auditory evoked re- sponses.^28,29^ The high prevalence of disorders reported in the literature when using the auditory processing paradigm through long latency potentials, in conjunction with our findings, suggests that epileptic patients may have sound processing deficits. Several authors,^31-33^ in their studies with epileptic children, have reported on the importance of areas such as the hippocampus, thalamus, cortical-thalamic circuit, temporal lobe and auditory association cortex in sound processing. Rei & Bao-Tian (1988)^30^ suggested that cognitive potential may originate from the thalamus or mesencephalon. In our study, there is a suggestion of impairment of auditory cortical functioning, which might have resulted from cerebral dysfunction and/or, in patients with partial crises, from the maturation process of the central nervous system.^34^ In addition, other variables such as the type of medication, prognosis and onset of seizures, which may interfere with cortical functioning, are worth mentioning.^21,31^ These provide evidence to suggest that further research is needed in order to investigate the correlation between auditory processing disorders and these factors.

## CONCLUSIONS

After applying and analyzing the SSW test among epileptic patients with partial and generalized crises we could conclude:

1. The majority of epileptic patients showed central auditory processing disorders in the SSW test.
2. There were no differences in relation to the partial or generalized crisis types. The two groups displayed similar performances, although the results from epileptic patients were quite different from the results obtained with normal subjects.
3. There were no significant differences in relation to test conditions. All possible disorder classifications were found, without the predominance of any specific disorder for either of the groups studied.

## References

[1] Broadbent DE (1954). The role of auditory localization in attention and memory span. J Exp Psychol.

[2] Kimura D (1963). A note on cerebral dominance in hearing. Acta Otolaryngol.

[3] Bellis TJ (1996). Assessment and management of central auditory processing disorders in the educational setting: from science to practice.

[4] Pereira LD, Pereira LD, Schochat E (1997). Processamento auditivo central: abordagem passo a passo. Processamento auditivo central: manual de avaliação.

[5] Giordani B, Berent S, Sackellares C (1985). Intelligence test performance of patients with partial and generalized seizures. Epilepsia.

[6] Smith DB, Craft BR, Collins J, Mattson RH, Cramer J (1986). Behavioral characteristics of epilepsy patients compared with normal controls. Epilepsia.

[7] Aldenkamp AP, Alpherts WCJ, Blennow G (1993). Withdrawal of antiepileptic medication in children - effects on cognitive function. The multicenter Holmfrid study. Neurology.

[8] Santos MFC (1993). Audiometria verbal: teste de reconhecimento de dissílabos através de tarefa dicótica.

[9] Câmara CC (1998). Teste de escuta dicótica de dissílabos (SSW) em crianças com e sem evidências de problemas escolares e/ou alterações das habilidades auditivas.

[10] Katz J, Ivey GR, Laufer W, Gabbay MS, Saralyn G, Katz J (1994). Spondaic procedures in central testing. Handbook of clinical audiology.

[11] Borges ACLC (1986). Adaptação do teste SSW para a língua portuguesa. Nota preliminar. Acta AWHO.

[12] Matsumoto H, Ajmone-Marsan C (1964). Cortical cellular phenomena in experimental epilepsy: interictal manifestations. Exp Neurol.

[13] Engel Jr J, Brown WJ, Kuhl DE, Phelps ME, Mazziotta JC, Crandall PH (1982). Pathological findings underlying focal temporal lobe hypometabolism in partial epilepsy. Ann Neurol.

[14] Cherlow DG, Dymond AM, Crandall PH, Walter RD, Serafetinides EA (1977). Evoked response and after-discharge thresholds to electrical stimulation in temporal lobe epileptics. Arch Neurol.

[15] Meldrum BS, Brierley JB (1972). Neuronal loss and gliosis in the hippocampus following repetitive epileptic seizures induced in adolescent baboons by allylglycine. Brain Res.

[16] Schwartzkroin PA, Prince DA (1978). Cellular and field potential properties of epileptogenic hippocampal slices. Brain Res.

[17] McLardy T (1969). Ammonshorn pathology and epileptic dyscontrol. Nature.

[18] Dymond AM, Crandall PH (1976). Oxygen availability and blood flow in the temporal lobes during spontaneous epileptic seizures in man. Brain Res.

[19] Engel J, Rausch R, Lieb JP, Kuhl DE, Crandall PH (1981). Correlation of criteria used for localization epileptic foci in patients considered for surgical therapy of epilepsy. Ann Neurol.

[20] Gevins AS, Zeitlin GM, Doyle JC (1979). Electroencephalogram correlates of higher cortical functions. Science.

[21] Musiek FE, Pinheiro ML (1987). Frequency pattern in cochlear, brainstem and cerebral lesions. Audiology.

[22] Deonna TW, Roulet E, Fontan D, Marcoz JP (1993). Speech and oromotor deficits of epileptic origin in benign partial epilepsy of childhood with rolandic spikes. Relationship to the acquired alphasia-epilepsy syndrome. Neuropediatrics.

[23] Staden U, Isaacs E, Boyd SG, Brandl U, Neville BG (1998). Language dysfunction in children with rolandic epilepsy. Neuropediatrics.

[24] Glowinski H (1973). Cognitive deficits in temporal lobe epilepsy: an investigation of memory functioning. J Nerv Ment Dis.

[25] Delaney RC, Rosen AJ, Mattson RH, Novelly RA (1980). Memory function in focal epilepsy: a comparison of non-surgical, unilateral temporal lobe and frontal lobe samples. Cortex.

[26] Farwell JR, Dodrill CB, Batzael LW (1985). Neuropsychological abilities of children with epilepsy. Epilepsia.

[27] Jambaque I, Dellatolas G, Dulac O, Ponsot G, Signoret JL (1993). Verbal and visual memory impairment in children with epilepsy. Neuropsychologia.

[28] Yingling CD, Skinner JE (1975). Regulation of unit activity in nucleus reticularis thalami by the mesencephalic reticular formation and the frontal granular cortex. Electroencephalogr Clin Neurophysiol.

[29] Fukai M, Motomura N, Kobayashi S, Asaba H, Sakai T (1990). Event-related potential (P300) in epilepsy. Acta Neurol Scand.

[30] Rei G, Bao-Tian F (1988). Diagnostic significance of the staggered spondaic word test and 40-Hz auditory event-related potentials. Audiology.

[31] Triantafyllou NI, Zalonis I, Kokotis P (1992). Cognition in epilepsy: a multichannel event-related potential (P300) study. Acta Neurol Scand.

[32] Kurthen M, Linke DB, Elger CE, Schramm J (1992). Linguistic perseveration in dominant-side intracarotid amobarbital tests. Cortex.

[33] Konishi T, Naganuma Y, Hongou K, Murami M, Yamatami M, Yagi S (1995). Changes of P300 latency with age in childhood epilepsy. Pediatr Neurol.

[34] Panayiotopoulos CP (1999). Benign childhood partial seizures and related epileptic syndromes.

